# Physicians’ perceptions regarding acute bleeding management: an international mixed qualitative quantitative study

**DOI:** 10.1186/s12871-021-01269-x

**Published:** 2021-02-10

**Authors:** Tadzio R. Roche, Doreen J. Wetli, Julia Braun, Ezequiel D. Kataife, Federico G. Mileo, Donat R. Spahn, David W. Tscholl, Sadiq Said

**Affiliations:** 1 Institute of Anesthesiology, University of Zurich and University Hospital Zurich, Raemistrasse 100, 8091 Zurich, Switzerland; 2 Departments of Epidemiology and Biostatistics, Epidemiology, Biostatistics and Prevention Institute, University of Zurich, Hirschengraben 84, 8001 Zurich, Switzerland; 3 Department of Anesthesiology, Hospital Italiano de Buenos Aires, Pres. Tte. Gral. Juan Domingo Peron 4190, C1199 Buenos Aires, Argentina

**Keywords:** Coagulation management, Acute bleeding, Decision making, Survey and questionnaires, Qualitative research, Diagnostics, Treatment, Algorithms

## Abstract

**Background:**

Acute bleeding is an omnipresent challenge for all physicians. Uncontrolled hemorrhage is the most common preventable cause of death after trauma worldwide. In different surgical disciplines, hemorrhage represents an independent risk factor for increased postoperative morbimortality, directly affecting patients’ outcomes. This study asked anesthesiologists about their personal perceived challenges when treating bleeding patients.

**Methods:**

This investigator-initiated, prospective, international, dual-center, mixed qualitative and quantitative study interrogated anesthesiologists about what they found easy and what difficult in treating acutely bleeding patients. Following the template approach for qualitative research, we identified major and minor topics through free inductive coding and word count. In a second step, we derived ten statements from the participants’ answers. Using a field survey, we then asked the participants to rate their level of agreement with the derived statements. We analyzed the answers using one sample Wilcoxon test and the Mann-Whitney test.

**Results:**

We included a total of 84 physicians in the qualitative interrogations and a different group of 42 anesthesiologists in the quantitative part. We identified 11 major topics and 19 associated subtopics. The main topics and the degree of agreement (here as agree or strongly agree) were as follows: “Complexity of the topic” (52.4% agreed to find the topic complex), “Cognitive aids” (92.9% agreed to find them helpful), “Time management” (64.3% agreed to feeling time pressure), “Human factors” (95.2% agreed that human factors are essential), “Resources” (95.2% agreed that resources are essential), “Experience” and “Low frequency of cases” (57.1% agreed to lack practice), “Diagnostic methods” (31.0% agreed that the interpretation of test results is difficult), “Anticoagulation” (85.7% agreed to it being difficult), “Treatment” (81.0% agreed to knowing the first therapeutic steps), and “Nothing”.

**Conclusions:**

Anesthesiologists in two large tertiary care facilities in different parts of the world found coagulation management, especially in anticoagulated patients, complex. We identified the delayed diagnostic test results and their interpretation as challenges. Resources, treatment protocols and human factors such as team communication were perceived to facilitate management. Future studies should explore the challenges in smaller hospitals and other parts of the world and test new technologies addressing the identified difficulties.

**Supplementary Information:**

The online version contains supplementary material available at 10.1186/s12871-021-01269-x.

## Background

Acute bleeding is a widespread challenge for all physicians, especially in surgical disciplines including anesthesiology and intensive care. Trauma continues to be a major cause of death worldwide [1], and uncontrolled hemorrhage is the most common preventable cause of death [2, 3]. A large, international, prospective cohort study reported severe bleeding as the most significant complication after noncardiac surgery, with hemorrhage being an independent risk factor for increased morbidity and mortality [4]. Also in cardiac surgery, major bleeding directly affects patients’ outcomes and causes significant additional costs of over 6′000 Euro [5].

Hence, effective hemostatic management is undoubtedly important. However, the optimal treatment of acute bleeding is still controversially discussed [6]. For example, the European guideline on the management of major hemorrhage after trauma favors viscoelastic-guided transfusion algorithms [7], while many large trauma centers in other parts of the world prefer predefined fixed ratio transfusion protocols [6, 8]. The multifactorial causes of bleeding [9, 10] and various methods of detecting coagulation disorders [11, 12] further complicate the understanding of this topic.

To the best of our knowledge, this is the first study to directly ask the treating physicians about the perceived challenges in coagulation management. We investigated the opinions of anesthesiologists in two different parts of the world, representing different sociocultural and economic circumstances. We hoped to identify common difficulties and shed light on the problems involved in treating acutely bleeding patients from a different perspective. Detecting these challenges may provide further incentives to address them through, for example, new technologies, improved approaches in education, or treatment plans.

## Methods

This was an investigator-initiated, prospective, international dual-center survey study. Using both qualitative and quantitative research methods, we explored the challenges anesthesiologists face in the treatment of acute bleeding. We included participants at the University Hospital Zurich, Switzerland, and the Italian Hospital of Buenos Aires in Argentina. These tertiary referral hospitals are regularly confronted with bleeding situations and therefore both have considerable experience in coagulation management. The Cantonal Ethics Committee of Zurich in Switzerland reviewed the study protocol and issued a declaration of no objection (Business Management System for Ethics Committees Number 2019–01090). The ethics committee responsible for the center in Buenos Aires approved the study protocol in a separate statement (N° 5357, dated November 14th, 2019). Before participation, we obtained written informed consent from each physician to use the collected data for research purposes.

### Qualitative part of this study

In the first part of this study, we conducted qualitative surveys. The choice of an appropriate sample size in qualitative research is an area of controversy and great uncertainty [13]. We applied a pragmatic approach for our study. We included the same 84 anesthesiologists of a previously published study that evaluated the Hemostasis Traffic Light, a decision support tool for acute coagulation management [14]. This allowed us to question the participants without much additional effort for them. In an undisturbed environment, we interrogated 21 residents and 21 staff physicians in each center. First, we obtained demographic data such as gender, age and job description. We then had the participants rate their skills in viscoelastic guided coagulation management on a scale from beginner (= 0) to expert (= 100). Beginning the open-ended interrogation, we asked the following two opposing questions to explore the full positive and negative experiences with the topic: What do you find easy and what do you find difficult in coagulation management? These questions aimed to identify both personal perceptions as well as external factors that influence the therapeutic process. There was no further guidance from the investigators. With no time limit, we encouraged all physicians to speak freely. We did not record the interrogations, but we simultaneously captured the answers as field notes on an iPad (Apple Inc.). Before submitting the final answers, we asked the participants whether they agreed with the collected field notes or whether they would like to change or add anything else.

**Fig. 1:**
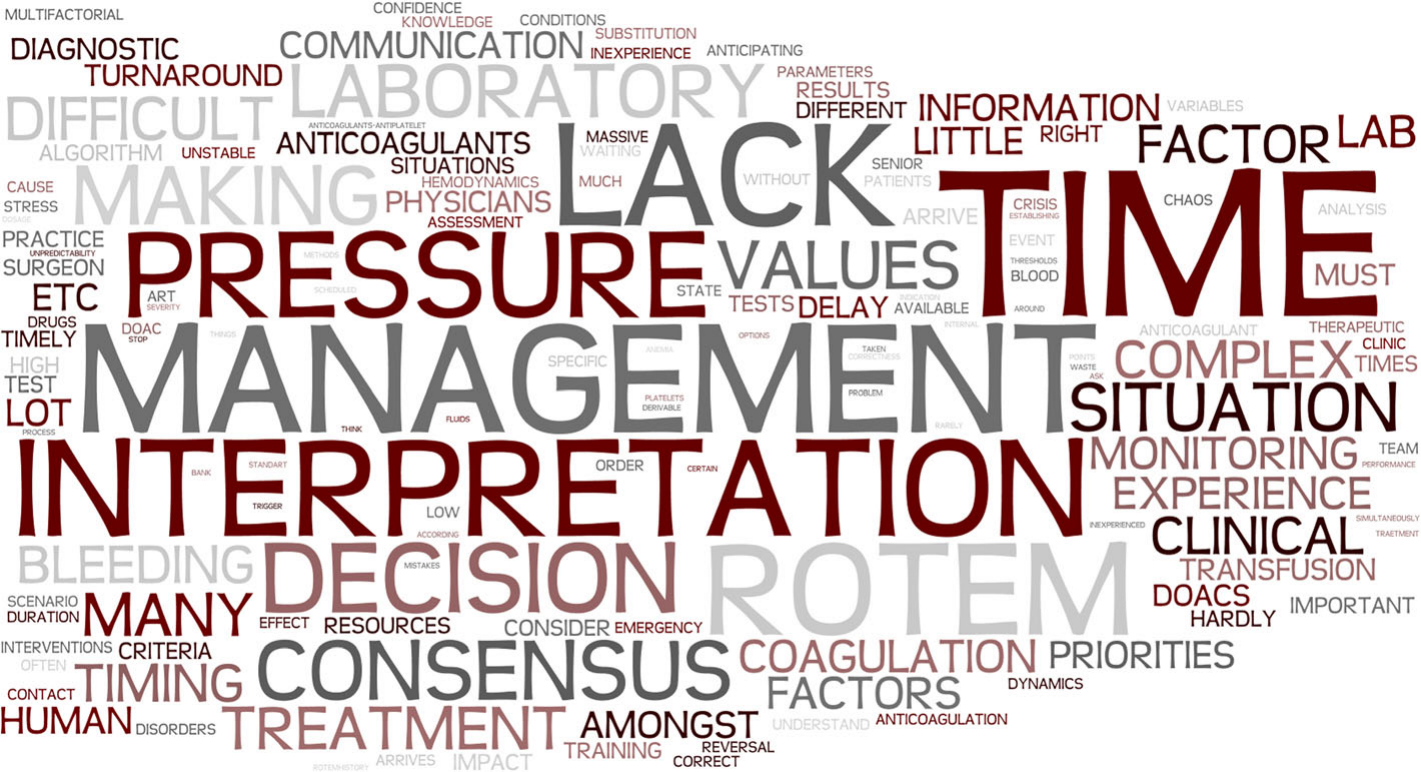
Graphical representation (Wordle.net) of the most common topics in the participants’ field notes. The word cloud makes more frequently used words appear larger. This cloud represents the answers to the question: “What do you find difficult in coagulation management?”

**Fig. 2:**
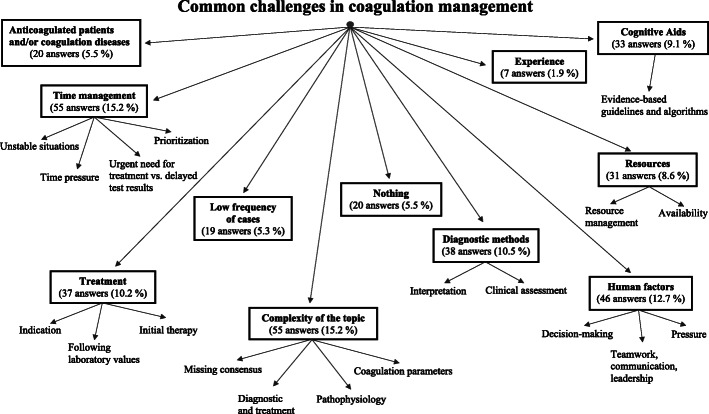
Graphical representation of the coding tree generated based on participants’ field note responses using word count and free inductive coding. The squared boxes indicate the 11 main topics, while the associated 19 subtopics are visualized by arrows. The participants gave a total of 361 answers

Beginning our qualitative analysis, we first translated the original answers from German and Spanish to English using Deepl.com (DeepL GmbH, Cologne, Germany). In Additional file 1, we provide the translated field notes. To analyze these translated data, we used the template approach [15]. This highly structured method for qualitative research begins with developing a coding template using the textual data set. It allows to predefine themes that are likely to be relevant to the research question. In our data set, we identified major topics with hierarchical subthemes based on word count using Microsoft Word (Microsoft Corporation, Redmond, WA, USA) and free inductive coding based on recurring participants’ answers. Figure 1 displays a quantitative graphical representation of the most common words in the field note responses. We modified the initial coding template until we agreed upon a final version, which, in our opinion, allowed a good representation of our data set. Complying with the criteria for reporting qualitative research [16], study authors TRR and DJW then independently applied this final coding template to our complete, translated data set. In case of disagreement between the two raters, we jointly determined a code for the respective answer. Figure 2 displays the final coding template.

### Quantitative part of this study

An additional, quantitative analysis of qualitative research can help generalize and supplement specific observations [17]. For the second part of this study, we derived ten statements from the main topics identified in our qualitative analysis. In each study center, the investigators randomly asked a new group of 21 anesthesiologists that did not participate in the previous qualitative part of the study, to rate these statements on a five-point Likert scale ranging from “strongly disagree” to “strongly agree”. We created the online survey using SurveyMonkey (SVMK Inc., San Matteo, CA, USA) and presented it on an Apple iPhone (Apple Inc., Cupertino, CA, USA). Participation was voluntary and we informed the physicians that it will take about 2 min to complete the survey. In Additional file 2, we provide the ten statements used for the quantitative part of this study.

### Statistical analysis

#### Qualitative analysis

We report the qualitative part of this study as number of coded responses and as percentage of all responses. We used Microsoft Excel (Microsoft Corporation) and Microsoft Word to manage our data. To assess how consistent the two study authors TRR and DJW implemented the generated coding template to the translated field notes, we calculated percent agreement and inter-rater reliability with Cohen’s kappa using R version 3.6.2 (R Foundation for Statistical Computing, Vienna, Austria) [18]. Further, we used Wordle.net to create a word cloud displaying the most commonly used words in the translated field notes.

### Quantitative analysis

We present the survey results of the quantitative part of this study as medians with interquartile ranges. Further, we used the Wilcoxon signed ranked test for one sample to test if the median differed from a neutral rating of each statement. Moreover, we used the Mann-Whitney test to investigate rating differences between the two study centers. We considered *p*-values under 0.05 to indicate statistical significance. This quantitative assessment aimed to achieve greater insight into the meaning of obtained qualitative results. We used R version 3.6.2 to analyze the quantitative data.

## Results

### Qualitative analysis

We performed the qualitative part of this study between January 92,020 and May 122,020. We interrogated 21 resident and 21 staff anesthesiologists in each study center, making it a total of 84 physicians. Regarding the gender distribution, 40.5% (34 of 84) were female. The participants displayed an overall work experience median (IQR [range]) of 5.0 (2.0–10.0 [0.0–30.0]) years. On a scale from beginner (= 0) to expert (= 100), the physicians rated their viscoelastic rotational thromboelastometry (ROTEM) skills median 40.0 (15.0–60.0 [0.0–100.0]).

Figure 1 and Additional file 3 display a graphical representation of the most common words in the field note responses. Using word count and free inductive coding, we generated the coding tree illustrated in Fig. 2. We identified 11 major topics with 19 associated subtopics in field note answers using both asked questions. We identified a total of 361 codes from the participants’ answers. The most frequently mentioned challenges in acute coagulation management included the complexity of this topic (55 of 361 answers, 15.2%), time pressure during the treatment (55 of 361 answers, 15.2%), human factors associated challenges such as communication, teamwork, or leadership (46 of 361 answers, 12,7%) and the challenging interpretation of diagnostic test results (38 of 361 answers, 10.5%). The physicians’ considered cognitive aids, such as coagulation algorithms (33 of 361 answers, 9.1%) and resources, such as personnel and blood products’ availability (31 of 361, 8.6%) to help in the treatment process. Table 1 lists the major topics identified, including participant examples. After the first round of coding and before agreeing on the final codes, inter-rater reliability between the two study authors TRR and DJW was strong with a percentage agreement of 86.15% with a Cohen’s kappa of 0.85.

**Table 1:** «What is easy/difficult about coagulation management»? Major topics with statement counts, percentages and examples. *ROTEM* rotational thromboelastometry

Major topics	Examples
Complexity of the topic (55 of 361 statements, 15.2%)	Participant #10: Pathophysiological relationships are difficult to understand. Participant #48: Much information to process.
Time management (55 of 361 statement, 15.2%)	Participant #8: Time factor waiting for values versus acute situation makes it difficult. Participant #40: Life threatening situation where time pressure is evident.
Human factors (46 of 361 statements, 12.7%)	Participant #3: Pressure that it is important for patients. Participant #66: Communication with surgeons and senior anesthesiologists.
Diagnostic methods (38 of 361 statements, 10.5%)	Participant #44: Assessment of the clinical situation. Participant #80: ROTEM management and interpretation.
Treatment (37 of 361 statements, 10.2%)	Participant #2: Initial measures are clear. Participant #30: Management of initial treatment (warming, volume replacement, etc.).
Cognitive aids (33 of 361 statements, 9.1%)	Participant #9: Clear guidelines help. Participant #15: A good algorithm makes management easy.
Resources (31 of 361 statements, 8.6%)	Participant #34: Resources available? Participant #47: Availability of the products.
Nothing (20 of 361 statements, 5.5%)	Participant #7: Nothing, it is difficult.
Anticoagulated patients and/or coagulation diseases (20 of 361 statements, 5.5%)	Participant #77: Antiplatelet drugs-related bleeding.
Low Frequency of Occurrence (19 of 361 statements, 5.3%)	Participant #14: Little contact with it.
Experience (7 of 361 statements, 1.9%)	Participant #1: Exercise improves management.

### Quantitative analysis

In the quantitative part of this study, we directly asked 11 residents and 10 staff anesthesiologists in each center to complete the survey, making it a total of 42 participants. The survey participation and completion rate were 100% (42 of 42, respectively). Regarding the gender distribution, 35.7% (15 of 42) were female. The overall work experience in years was median 4.2 (2.8–8.0 [0.3–33.0]) and the participants rated their ROTEM skills median 50.0 (30.0–64.8 [0.0–100.0]).

**Fig. 3:**
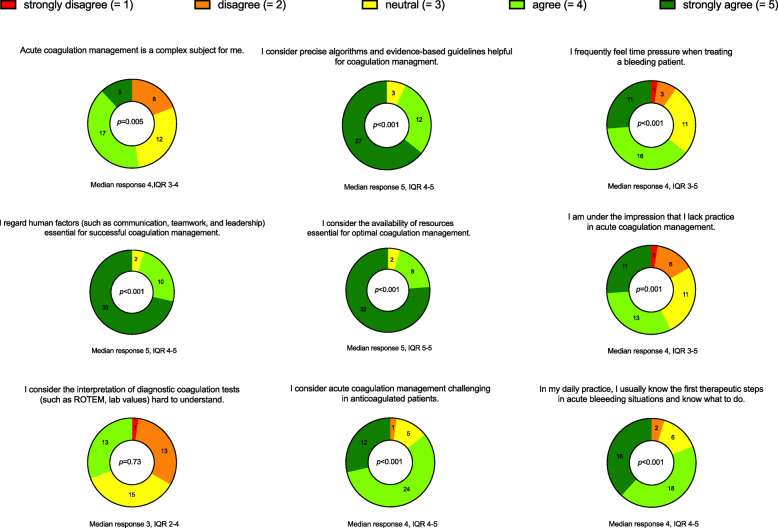
The field survey results of the participant ratings as part-of-whole donut charts. We provide the nine statements with median and interquartile (IQR) ranges. To counteract the problem of multiple comparisons, we regarded a p-value of 0.0025 to indicate statistically significant difference from neutral. ROTEM = rotational thromboelastometry. *n* = 42

The first derived statement asked the participants to rate the difficulty of coagulation management on a scale from very difficult (=1) to very easy (=5). The median response was 2, with an IQR from difficult (=2) to neutral (=3). Figure 3 displays the rating results of the nine statements derived from the qualitative analysis of this study. To counteract the problem of multiple comparisons, we applied Bonferroni correction. Hence, we divided the original level of significance by 20, the number of tests, and regarded a *p*-value of 0.0025 to indicate significant difference for these comparisons. Mann-Whitney test yielded no significant difference between the centers in rating each of the statements. We provide the full quantitative analysis in Additional file 2.

## Discussion

In this mixed qualitative and quantitative study, we identified the perceived challenges of anesthesiologists in treating acute bleeding. Mentioned topics included the complexity of this topic, time pressure during the treatment, human factors associated challenges, the difficulty in managing anticoagulated patients, and the challenging interpretation of diagnostic test results. However, the physicians considered cognitive aids such as coagulation algorithms and resources such as the availability of personnel and blood products to help in the treatment process.

Based on the interrogation field note responses, we identified the complexity of acute bleeding management as a major challenge with subthemes such as complex algorithms or difficult pathophysiological understanding. Several factors such as hypovolemic shock or thrombin generation contribute to the complexity of coagulopathy [2, 9], and others such as the presence of anticoagulants, hypothermia, or hemodilution deteriorate the coagulopathic condition [19, 20]. These interactions of different patient factors as well as external factors render it difficult for the clinician to maintain an optimal overview. In our quantitative assessment, only 19.1% (8 of 42) disagreed with the statement that acute coagulation management is complex, and 92.9% (39 of 42) considered algorithms and guidelines helpful. Analyzing the field notes, we also identified cognitive aids as important tools to facilitate coagulation management. Our results align with other research authors’ opinions and highlight the importance of comprehensive medical training and clearly defined treatment algorithms. The authors of the European Trauma Treatment Guidelines, for example, believe that education and adhering to coagulation protocols yield the greatest improvement in patient outcomes [7]. Other studies showed that the implementation of such coagulation algorithms indeed reduced patient mortality significantly [21, 22]. Further, a user-centered design of the treatment protocols also had significant positive effects with seven times higher odds ratios of correctly treating a simulated bleeding case [14]. To best manage these stressful bleeding situations, we should strive to promote and simplify our medical staff’s training and provide well-structured, institutional coagulation algorithms.

We identified time management as another main challenge, with subthemes such as time pressure and rapidly changing situations. In our quantitative analysis, 64.3% (27 of 42) of the physicians agreed or strongly agreed that they feel time pressure when treating a bleeding patient and 26.2% (11 of 42) rated the same statement as neutral. Since laboratory-based clotting tests may require over an hour [23], their utility for acute treatment is limited [24]. Delayed diagnosis and therapy can lead to further complications of bleeding [25]. Bedside point-of-care devices such as viscoelastic test methods have accelerated the time to assess the coagulation status [26, 27]. However, even though viscoelastic testing is routinely applied in both study centers, the University Hospital Zurich and the Italian Hospital of Buenos Aires, the participating physicians still perceived time management as a major challenge in treating bleeding patients.

In our qualitative analysis, we found that both resources and human factors with subtopics such as teamwork and communication are considered main topics in acute coagulation management. In the field survey, 95.2% (40 of 42) of the anesthesiologists agreed with the statement that they regard human factors and resources such as the availability of personnel and blood products essential for the successful treatment of bleeding patients. Further, 57.1% (24 of 42) agreed that they feel they lack practice in bleeding management. Although the participants were anesthesiologists in tertiary care facilities, these results indicate that resources, human factors and training are considered important despite the size of the hospital and the area of specialization. Simulated environments may provide a setting to gain training, experience and confidence in bleeding management, focusing on human factors such as team performance [28, 29].

Furthermore, diagnostic methods were also perceived as a main challenge in treating hemorrhagic patients. Some participants mentioned the interpretation of viscoelastic tests such as ROTEM as difficult. Regarding the survey, 31.0% (13 of 42) of the physicians agreed to the statement that diagnostic coagulation tests are hard to understand and another 35.7% (15 of 42) rated neutral. Our research group identified the interpretation of diagnostic test results as a problem several years ago, and hence created a first attempt to simplify viscoelastic test outputs. We created Visual Clot technology, an animated blood clot that displays ROTEM results in a user-centered design [30]. This simplification helped the participating anesthesiologists from Switzerland and Germany to solve simulated bleeding scenarios faster, with more therapeutic confidence and less workload [30]. The field note responses of the current study support our opinion that there is a need to simplify the interpretation of viscoelastic testing.

Finally, we identified anticoagulation as a main topic, and 85.7% (36 of 42) of the anesthesiologists agreed with the statement that acute coagulation management is especially challenging in anticoagulated patients. Since antithrombotic drugs decrease thromboembolic events [31] and the newer direct oral anticoagulants are easier to monitor [32], they have become some of the most commonly prescribed medications [33]. As treating physicians, we are now faced with the challenge of adapting our therapeutic options to these rapid changes in anticoagulation therapy. All the more effort should now be put into teaching our clinical staff.

### Strengths and limitations

The qualitative part of this study has an inherent limitation. In all qualitative research, every observation receives the same attention regardless of its frequency or chance to occur. Hence, the qualitative results obtained cannot be extrapolated with certainty to a wider population [34]. However, this study also possesses several strengths. The international, dual-center design helped to identify a broader range of opinions on the challenges in acute coagulation management. Further, anesthesiologists in tertiary care hospitals are best suited to explore this study question as they are regularly confronted with acute bleeding situations. The participants of this study were randomly selected according to their availability during clinical practice. Finally, following up qualitative research with a quantitative assessment helped to achieve greater insight into the meaning of obtained main topics.

## Conclusions

This dual-center study gave us insight into the impressions of clinical anesthesiologists regarding acute bleeding management in two different parts of the world. To optimize the care of bleeding patients, we should focus on facilitating the understanding of the complex pathophysiological interactions within the clotting system, including the effects of anticoagulants, and the interpretation of diagnostic tests. Further, we should strive designing strategies to improve timely management of diagnostic and therapeutic resources. Finally, we should promote the availability of institutional coagulation algorithms more as physicians found them valuable for medical practice. We recommend that ideal coagulation algorithms and diagnostic test outputs should be user-centered and readily available. Future qualitative research should explore the opinions in smaller health care facilities and other parts of the world. Quantitative research should evaluate the effects of interventions aimed at improving the quality of coagulation management.

## Supplementary Information

**Additional file 1.** Translated participants’ field notes.

**Additional file 2.** Statements of the quantitative part of this study, including full quantitative analysis.

**Additional file 3.** Graphical representation of the field notes answers to the question: “What do you find easy in coagulation management?”.

## Data Availability

The datasets supporting the conclusions of this article are available in this article and its supplementary information files.

## Abbreviations

IQR: interquartile range
ROTEM: rotational thromboelastometry
