# Supplementary material for: Physicians’ perceptions regarding acute bleeding management: an international mixed qualitative quantitative study
Source: BMC Anesthesiol. 2021 Feb 10;21:43. doi: 10.1186/s12871-021-01269-x (PMC7874660; doi:10.1186/s12871-021-01269-x)
Supplement: Supplementary file 1 — Additional file 1. Translated participants’ field notes. [file 12871_2021_1269_MOESM1_ESM.pdf]

Step back and reassess – What physicians see as challenges in treating patients with acute bleeding: international, mixed qualitative and quantitative study

Tadzio R. Roche[1], Doreen J. Wetli[1], Julia Braun[2], Ezequiel D. Kataife[3], Federico G. Mileo[3], Donat R. Spahn[1], David W. Tscholl[1,*], Sadiq Said[1]

Additional file 1 - All translated participants' field notes

This document contains all translated field notes of the coagulation management survey study. We translated the answers using Deepl.com (DeepL GmbH, Cologne, Germany). The answers listed are anonymous.

Participant 1:

Question 1: What is difficult about coagulation management?

[Interpretation of ROTEM]
[Clinic and laboratory differ greatly]

Question 2: What is easy about coagulation management?

[Exercise improves management]
[Surgical hemostasis is the easiest]

Participant 2:

Question 1: What is difficult about coagulation management?

[A lot of information]
[Fear of making mistakes]
[Dosage]

Question 2: What is easy about coagulation management?

[Initial measures are clear]

Participant 3:

Question 1: What is difficult about coagulation management?

[Lack of experience]
[Hardly any overview of the laboratory parameters, difficult to find your way around]
[Interpretation of the parameters is difficult]
[Pressure that it is important for patients]

Question 2: What is easy about coagulation management?

[Clear guidelines with limit values]

Participant 4:

Question 1: What is difficult about coagulation management?

[-]

Question 2: What is easy about coagulation management?

[Simple if you have algorithm in mind]

Participant 5:

Question 1: What is difficult about coagulation management?

[Coagulation management is complex]

Question 2: What is easy about coagulation management?

[-]

Participant 6:

Question 1: What is difficult about coagulation management?

[Different pathways and many factors]
[Hardly any ROTEM experience]

Question 2: What is easy about coagulation management?

[Wrong therapy has little effect]
[Benefits are greater than disadvantages]

Participant 7:

Question 1: What is difficult about coagulation management?

[Effect of anticoagulants on specific factors]
[Implementation of the internal directive]

Question 2: What is easy about coagulation management?

[Nothing, it's difficult. Maybe with practice, it'll be easy.]

Participant 8:

Question 1: What is difficult about coagulation management?

[Complex, many factors to consider.]
[Time factor waiting for values versus acute situation makes it difficult.]

Question 2: What is easy about coagulation management?

[Initial measures are simple.]
[The cornerstones are clear.]

Participant 9:

Question 1: What is difficult about coagulation management?

[Experience.]
[Interpretation of Rotem.]

Question 2: What is easy about coagulation management?

[Clear guidelines help]

Participant 10:

Question 1: What is difficult about coagulation management?

[Pathophysiological relationships are difficult to understand.]
[A lot to memorize.]
[Not derivable.]

Question 2: What is easy about coagulation management?

[Read algorithm.]
[Basic measures are clear.]

Participant 11:

Question 1: What is difficult about coagulation management?

[Time pressure in unstable situations.]
[A lot of information must be collected.]
[Interpretation of the coagulation system.]
[Pre-treatment with anticoagulants.]

Question 2: What is easy about coagulation management?

[Algorithm gives clear definitions.]
[Distribution of work.]

Participant 12:

Question 1: What is difficult about coagulation management?

[Timely correct interpretation of the ROTEM is difficult.]
[In general, the time management is also demanding.]

Question 2: What is easy about coagulation management?

[Interpretation of the usual parameters and correction of these is easy.]
[Implementation of the basic measures.]

Participant 13:

Question 1: What is difficult about coagulation management?

[Lack of practice.]
[Under time pressure.]
[Many information at the same time]

Question 2: What is easy about coagulation management?

[Therapy clear when problem/deficiency is diagnosed]

Participant 14:

Question 1: What is difficult about coagulation management?

[Very complex to understand in theory.]
[Little contact with it.]

Question 2: What is easy about coagulation management?

[Nothing, it's difficult.]

Participant 15:

Question 1: What is difficult about coagulation management?

[Inexperienced.]
[Very high clinical time pressure.]
[High scope of the decisions.]

Question 2: What is easy about coagulation management?

[A good algorithm makes management easy.]
[Decision making is discussed in the team, so agreements are possible.]

Participant 16:

Question 1: What is difficult about coagulation management?

[to identify the cause of the bleeding.]
[to know reference values.]
[Inexperience with ROTEM.]
[History of the patient must be taken into account (e.g. anticoagulation).] [Challenging.]

Question 2: What is easy about coagulation management?

[Experience of values simplifies their application.]

Participant 17:

Question 1: What is difficult about coagulation management?

[Complex.]
[Many points to think about]

Question 2: What is easy about coagulation management?

[-]

Participant 18:

Question 1: What is difficult about coagulation management?

[hardly any contact as a beginner.]
[Time pressure.]
[Impact of the decision.]
[Very complex subject.]

Question 2: What is easy about coagulation management?

[Nothing.]

Participant 19:

Question 1: What is difficult about coagulation management?

[Algorithm must be repeated.]
[There is a lot to consider overall.]

Question 2: What is easy about coagulation management?

[Algorithm helps in stressful situations.]

Participant 20:

Question 1: What is difficult about coagulation management?

[Interpretation of ROTEM analysis]

Question 2: What is easy about coagulation management?

[-]

Participant 21:

Question 1: What is difficult about coagulation management?

[Often not all information available.]
[Identifying the problem is difficult.]
[Little experience.]
[Little training.]
[Complex.]

Question 2: What is easy about coagulation management?

[Algorithms makes it easy.]
[Action is predetermined.]
[Information.]

Participant 22:

Question 1: What is difficult about coagulation management?

[Criteria and decision making]

Question 2: What is easy about coagulation management?

[-]

Participant 23:

Question 1: What is difficult about coagulation management?

[delayed lab results.]
[Fast dynamics.]
[Unstable situations.]

Question 2: What is easy about coagulation management?

[Algorithms; defined order.]
[ROTEM as fast response.]
[Basic actions are clear.]

Participant 24:

Question 1: What is difficult about coagulation management?

[many variables.]
[Lack of knowledge.]
[Different measurement methods]

Question 2: What is easy about coagulation management?

[Nothing]

Participant 25:

Question 1: What is difficult about coagulation management?

[Differentiate what is important what is less important]

Question 2: What is easy about coagulation management?

[-]

Participant 26:

Question 1: What is difficult about coagulation management?

[Substitution without values]

Question 2: What is easy about coagulation management?

[-]

Participant 27:

Question 1: What is difficult about coagulation management?

[DOACs management.]
[What type of monitoring to use for each clinical scenario and according to the senior anaesthesiologist]

Question 2: What is easy about coagulation management?

[initial management]
[fluidtherapy]
[fibrinogen replacement]

Participant 28:

Question 1: What is difficult about coagulation management?

[The amount of variables to manage at the same time]
[Cognitive overload]
[Time pressure]
[Human factor]
[Low frequency of the event]
[Lack of training]

Question 2: What is easy about coagulation management?

[Monitoring tools availability]

Participant 29:

Question 1: What is difficult about coagulation management?

[When special situations occur (DOACs management, congenital disease, hidden bleeding)]
[time pressure]
[lack of consensus amongst physicians]
[timing for interventions]
[clinical assesment]

Question 2: What is easy about coagulation management?

[-]

Participant 30:

Question 1: What is difficult about coagulation management?

[ROTEM, as a monitoring, admitted lack of study.]
[Balancing fluids and coagulation management.]
[Chaos situation, lack of consensus and guidance for management of the situation.]
[Human factor in terms of team working (communication, performance, leadership, etc)]

Question 2: What is easy about coagulation management?

[baseline conditions management (warming, volume reposition, etc).]

Participant 31:

Question 1: What is difficult about coagulation management?

[lack of consensus]
[not following any guideline or algorithm]
[state of the art management]
[human factor]
[algorithms not adapted to the users or their resources]

Question 2: What is easy about coagulation management?

[The mere existence of algorithm should make it easy, read and execute.]

Participant 32:

Question 1: What is difficult about coagulation management?

[monitoring criteria for Lab test (when to ask i.e. AntiXa, ROTEM, etc.)]
[therapeutic resources administration, a lot of options available.]

Question 2: What is easy about coagulation management?

[the presence of an algorithm to follow helps a lot.]
[not hard to clinically assess a bleeding.]

Participant 33:

Question 1: What is difficult about coagulation management?

[specific coagulation disorders management]
[resources management]
[human factor]
[anticoagulants management (Xabans and antiII)]

Question 2: What is easy about coagulation management?

[monitoring lab and clinical assessment]

Participant 34:

Question 1: What is difficult about coagulation management?

[Time pressure and delay of the analysis]

Question 2: What is easy about coagulation management?

[Algorithm with clear structured procedure]
[fast diagnostics via point of care laboratory]
[Resources available]
[enough staff]

Participant 35:

Question 1: What is difficult about coagulation management?

[Many factors must be interpreted simultaneously]

Question 2: What is easy about coagulation management?

[Algorithmus]
[Labor]
[Rotem (point of care)]

Participant 36:

Question 1: What is difficult about coagulation management?

[anticoagulants-antiplatelet management (emergency bleeding patients on these drugs)]
[communication amongst physicians in the team]
[when to stop a massive transfusion]

Question 2: What is easy about coagulation management?

[Hierarchy]
[Treatment priorities]
[Leadership comes easy based on training and knowledge.]

Participant 37:

Question 1: What is difficult about coagulation management?

[Duration until laboratory values arrive]
[Anticipating the course of bleeding]

Question 2: What is easy about coagulation management?

[Clearly defined]
[Experience]

Participant 38:

Question 1: What is difficult about coagulation management?

[Impact of the decision]
[Time until the laboratory arrives]

Question 2: What is easy about coagulation management?

[Many resources]

Participant 39:

Question 1: What is difficult about coagulation management?

[Lack of consensus amongst people]
[if correct treatment was administered, confidence in treatment]
[Chaos situation]
[Establishing appropriate order, treatment priorities]

Question 2: What is easy about coagulation management?

[Following lab values]
[There are enough resources (diagnostic/therapeutics)]
[Having algorithms]

Participant 40:

Question 1: What is difficult about coagulation management?

[they are life threatening situation, where time pressure is evident]
[high stress levels for decision making]
[lack of consensus]
[the absence of tangible elements to treat in the clinical field (factors, platelets, etc)]
[lack of experience / knowledge on certain fields]

Question 2: What is easy about coagulation management?

[the possibility to generate a stepwise/protocolised approach to a complex scenario]

Participant 41:

Question 1: What is difficult about coagulation management?

[the different complex scenarios (emergency, trauma, scheduled surgery, etc)]
[treatment priorities, where to begin]
[lack of consensus generating waste of time]

Question 2: What is easy about coagulation management?

[When we have expected situations (known patient history, high risk bleeding procedures)]

Participant 42:

Question 1: What is difficult about coagulation management?

[Time until laboratory values arrive.]

Question 2: What is easy about coagulation management?

[Initial measures are clearly defined.]
[Rotem and Point of care Laboratory makes it easy.]

Participant 43:

Question 1: What is difficult about coagulation management?

[Monitoring data on time (ROTEM, LAB, ETC)]
[Decision making balanced on clinical assessment rather than analytical]
[Lack of consensus amongst physicians]
[Anemia treatment and transfusion triggers.]

Question 2: What is easy about coagulation management?

[Tranexamic acid indication]
[Fibrinogen concentrate indication]
[Situational awareness]
[Resources management]

Participant 44:

Question 1: What is difficult about coagulation management?

[Time delay due to laboratory values.]
[Assessment of the clinic]

Question 2: What is easy about coagulation management?

[Clear guidelines.]
[Algorithm]

Participant 45:

Question 1: What is difficult about coagulation management?

[Many factors to consider.]
[Impact.]
[Waiting on labs.]

Question 2: What is easy about coagulation management?

[Clear guidelines.]
[Point of care Laboratory.]

Participant 46:

Question 1: What is difficult about coagulation management?

[Effect with risk of thrombosis.]
[Duration of the laboratory values.]
[Low experience.]

Question 2: What is easy about coagulation management?

[Clear guidelines.]
[Point of care]
[Rotem.]

Participant 47:

Question 1: What is difficult about coagulation management?

[Estimate kinetics.]
[Time delay until laboratory arrives.]
[Availability of the products.]

Question 2: What is easy about coagulation management?

[Clear guidelines.]
[Great evidence of the measures.]

Participant 48:

Question 1: What is difficult about coagulation management?

[Much information to process, but standardized]

Question 2: What is easy about coagulation management?

[Much information to process, but standardized]

Participant 49:

Question 1: What is difficult about coagulation management?

[Time until laboratory values arrive]
[Indication for coagulation substitution]

Question 2: What is easy about coagulation management?

[Rotem]
[Algorithm]

Participant 50:

Question 1: What is difficult about coagulation management?

[Give the right medication at the right time.]
[Interpretation is difficult.]
[Time pressure.]

Question 2: What is easy about coagulation management?

[Rotem and point of care Laboratory.]
[Factor substitution.]

Participant 51:

Question 1: What is difficult about coagulation management?

[Results show outdated conditions.]
[Time delay of the laboratory.]
[Documentation of circumstances often insufficient.]

Question 2: What is easy about coagulation management?

[Nothing.]

Participant 52:

Question 1: What is difficult about coagulation management?

[Anticipating, estimating the dynamics.]
[Multifactorial.]
[Occurs rarely, therefore lack of practice.]

Question 2: What is easy about coagulation management?

[limited therapy options.]
[Teamwork.]

Participant 53:

Question 1: What is difficult about coagulation management?

[Time pressure.]
[Inexperience.]

Question 2: What is easy about coagulation management?

[Nothing.]

Participant 54:

Question 1: What is difficult about coagulation management?

[Order-priorization]
[Multiple posible treatments]

Question 2: What is easy about coagulation management?

[Preconditions are always the same.]

Participant 55:

Question 1: What is difficult about coagulation management?

[Decision making]
[Tests interpretation]
[Treatment timing (priorities)]

Question 2: What is easy about coagulation management?

[Nothing]

Participant 56:

Question 1: What is difficult about coagulation management?

[Transfusion thresholds]
[ROTEM interpretation]
[Treatment timming]

Question 2: What is easy about coagulation management?

[Cognitive aids]

Participant 57:

Question 1: What is difficult about coagulation management?

[Anticoagulant reversion (Noacs)]
[Abnormal results without bleeding]

Question 2: What is easy about coagulation management?

[Preconditions treatment are always the same]

Participant 58:

Question 1: What is difficult about coagulation management?

[Timely decision making]
[Test interpretation]
[Time pressure]

Question 2: What is easy about coagulation management?

[Nothing]

Participant 59:

Question 1: What is difficult about coagulation management?

[Timely interventions are difficult]
[Test interpretation]

Question 2: What is easy about coagulation management?

[Nothing]

Participant 60:

Question 1: What is difficult about coagulation management?

[Lab monitoring]
[right treatment]

Question 2: What is easy about coagulation management?

[cause diagnosis (surgical/coagulopathy)]

Participant 61:

Question 1: What is difficult about coagulation management?

[lack of consensus amongst physicians]
[anticoagulants]
[topic dislike]

Question 2: What is easy about coagulation management?

[Nothing]

Participant 62:

Question 1: What is difficult about coagulation management?

[little practice with ROTEM]
[reflective learning]
[lack of self confidence]
[lack of consensus or official algorithm]

Question 2: What is easy about coagulation management?

[Clinical patterns in certain scenarios (cardiac surgery, trauma)]

Participant 63:

Question 1: What is difficult about coagulation management?

[time pressure]
[clinical severity]
[decision correctness]
[lack of consensus]

Question 2: What is easy about coagulation management?

[ROTEM pattern visuals]
[having algorithms]

Participant 64:

Question 1: What is difficult about coagulation management?

[ROTEM interpretation]
[DOACs management]
[criteria between PCC and FFP]

Question 2: What is easy about coagulation management?

[hemodynamics]
[red blood cell and platelet therapy]

Participant 65:

Question 1: What is difficult about coagulation management?

[DOAC reversal management]
[hemodynamics]

Question 2: What is easy about coagulation management?

[leadership]
[communication]

Participant 66:

Question 1: What is difficult about coagulation management?

[ROTEM interpretation and decision making]
[Communication with surgeons and senior anaesthesiologists]
[Timing]
[stress management]
[DOAC management]
[Lab monitoring]
[Plasma transfusion trigger]

Question 2: What is easy about coagulation management?

[having an algorithm]
[having the resources]

Participant 67:

Question 1: What is difficult about coagulation management?

[ROTEM interpretation]

Question 2: What is easy about coagulation management?

[communication]
[decision making in known clinical settings]

Participant 68:

Question 1: What is difficult about coagulation management?

[ROTEM interpretation]

Question 2: What is easy about coagulation management?

[Comunication]
[clinical criteria]

Participant 69:

Question 1: What is difficult about coagulation management?

[timing for decision making]
[gap between diagnostic tests and clinical status]

Question 2: What is easy about coagulation management?

[factor concentrate therapy]
[target therapy]

Participant 70:

Question 1: What is difficult about coagulation management?

[massive haemorrhage where almost everything]
[ROTEM interpretation]
[anticoagulation management]

Question 2: What is easy about coagulation management?

[human factor and communication]
[liberty to decide treatment]

Participant 71:

Question 1: What is difficult about coagulation management?

[timing for decision making]
[diagnostic turnaround times]
[human factor organization and priorities]
[scenario and infrastructure situation]
[out of OR areas]
[training]

Question 2: What is easy about coagulation management?

[resources availability (diagnostic/therapeutic)]
[Team work]

Participant 72:

Question 1: What is difficult about coagulation management?

[surgeon pressure]
[communication breakdown]

Question 2: What is easy about coagulation management?

[resources management]
[teamwork]
[collective thinking amongst anaesthesia team]

Participant 73:

Question 1: What is difficult about coagulation management?

[timing turnaround for blood compoments]
[ROTEM interpretation]
[blood bank communication]
[surgeon underestimates the situation]

Question 2: What is easy about coagulation management?

[basic preconditions]

Participant 74:

Question 1: What is difficult about coagulation management?

[borderline values]
[grey zone]
[factor concentrate vs hemocomponentes approach]
[DOACs management]
[surgeon pressure]

Question 2: What is easy about coagulation management?

[clear algorithm strongly evidence based]

Participant 75:

Question 1: What is difficult about coagulation management?

[Tests interpretation]
[Traetment of pre existing conditions]

Question 2: What is easy about coagulation management?

[-]

Participant 76:

Question 1: What is difficult about coagulation management?

[the abscense of a protocol in a crisis situation]

Question 2: What is easy about coagulation management?

[ROTEM interpretation]
[type of bleeding recognition]

Participant 77:

Question 1: What is difficult about coagulation management?

[antiplatelet drugs related bleeding]

Question 2: What is easy about coagulation management?

[hypovolemic shock management and its coagulopathy]
[factor concentrate therapy]

Participant 78:

Question 1: What is difficult about coagulation management?

[lack of consensus or standart procedures]
[too much state of the art]
[too many things to do in little time]
[unpredictability of the situation]
[infrequent event]
[platelet function disorders]

Question 2: What is easy about coagulation management?

[it's hard to do harm when giving treatment]
[possible causes are usually repeated]

Participant 79:

Question 1: What is difficult about coagulation management?

[anticoagulant management]
[reversal]
[When I haven't got he ROTEM]

Question 2: What is easy about coagulation management?

[Following an algorithm and guided decision making]

Participant 80:

Question 1: What is difficult about coagulation management?

[diagnostic turnaround times for decision making]

Question 2: What is easy about coagulation management?

[Resource management]
[ROTEM management and interpretation]

Participant 81:

Question 1: What is difficult about coagulation management?

[therapeutic target through diagnostic tools]

Question 2: What is easy about coagulation management?

[Nothing.]

Participant 82:

Question 1: What is difficult about coagulation management?

[timing and coordination in hemodynamics and coagulation management]
[need for help]
[time pressure in crisis management]
[Priorities order]

Question 2: What is easy about coagulation management?

[decision making process coming from experience]
[communication with team members]

Participant 83:

Question 1: What is difficult about coagulation management?

[multifactorial bleeding cause]
[turnaround times from lab for decision making]

Question 2: What is easy about coagulation management?

[systematic, it is protocolised]

Participant 84:

Question 1: What is difficult about coagulation management?

[Much information to process]
[timing for decision making]

Question 2: What is easy about coagulation management?

[target therapy]
