# Supplementary material for: Physicians’ perceptions regarding acute bleeding management: an international mixed qualitative quantitative study
Source: BMC Anesthesiol. 2021 Feb 10;21:43. doi: 10.1186/s12871-021-01269-x (PMC7874660; doi:10.1186/s12871-021-01269-x)
Supplement: Supplementary file 2 — Additional file 2. Statements of the quantitative part of this study, including full quantitative analysis. [file 12871_2021_1269_MOESM2_ESM.pdf]

Step back and reassess – What physicians see as challenges in treating patients with acute bleeding: international, mixed qualitative and quantitative study

Tadzio R. Roche[1], Doreen J. Wetli[1], Julia Braun[2], Ezequiel D. Kataife[3], Federico G. Mileo[3], Donat R. Spahn[1], David W. Tscholl[1,*], Sadiq Said[1]

Additional file 2 – Quantitative results and statements

Statements used in the field survey:

Q1	How would you rate the difficulty of acute coagulation management?
Q2	Acute coagulation management is a complex subject for me.
Q3	I frequently feel time pressure when treating a bleeding patient.
Q4	I regard human factors (such as communication, teamwork, and leadership) essential for successful coagulation management.
Q5	I consider the interpretation of diagnostic coagulation tests (such as ROTEM, lab values) hard to understand.
Q6	I consider acute coagulation management challenging in anticoagulated patients.
Q7	I am under the impression that I lack practice in acute coagulation management.
Q8	I consider precise algorithms and evidence-based guidelines helpful for coagulation management.
Q9	In my daily practice, I usually know the first therapeutic steps in acute bleeding situations and know what to do.
Q10	I consider the availability of resources (e.g., diagnostic test facilities, sufficient staff availability) essential for optimal coagulation management.

Abbreviations and definitions used in the document:

Experience	Professional experience in years.
HIBA	Hospital Italiano de Buenos Aires.
Rating Q1:	1 =very difficult, 2=difficult, 3=neutral, 4=easy, 5=very easy
Ratings Q2 to Q10:	1=Strongly disagree, 2= Disagree, 3=Neutral, 4=Agree, 5=Strongly agree
ROTEM_per_year	Rated number of ROTEM interpreted per year.
ROTEM_skills	Self-rated ROTEM skills from 0 (=beginner) to 100 (=expert).
USZ	University Hospital Zurich.

Coagulation management

Version 1.0
Julia Braun

November 20, 2020

1 Descriptives

Variable	n	Min	q_1	$\widetilde{x}$	$\bar{x}$	q_3	Max	s	IQR	#NA
Experience	42	0.3	3	4.2	7.0	8.0	33	7.6	5.0	0
ROTEM_per_year	42	0.0	5	11.5	24.4	40.0	100	27.7	35.0	0
ROTEM_skills	42	0.0	30	50.0	48.3	64.8	100	26.6	34.8	0

Table 1: Descriptive table - continuous data, Experience = professional experience in years, ROTEM_per_year = ratet number of Rotational thrombelastometry (ROTEM) interpreted per year, ROTEM_skills = self-rated ROTEM skills from 0 (=beginner) to 100 (=expert).

Variable	Levels	n	%	$\sum$%
Center	USZ	21	50.0	50.0
	HIBA	21	50.0	100.0
	all	42	100.0	
Gender	Male	27	64.3	64.3
	Female	15	35.7	100.0
	all	42	100.0	
Profession	Resident	22	52.4	52.4
	Staff Physician	20	47.6	100.0
	all	42	100.0	

Table 2: Descriptive table - categorical data, Center = study center (USZ = University Hospital Zurich, HIBA = Hospital Italiano de Buenos Aires).

2 Medians of questions 1 to 10

Note that there are many tests in this analysis (10 times Wilcoxon). For this reason, Bonferroni correction should be applied for the specified p-values.

Variable	n	Min	q_1	$\tilde{x}$	q_3	Max	IQR	*p-value*
Q1	42	1	2	2	3.0	4	1.0	0.0021
Q2	42	2	3	4	4.0	5	1.0	0.0046
Q3	42	1	3	4	4.8	5	1.8	<0.001
Q4	42	3	4	5	5.0	5	1.0	<0.001
Q5	42	1	2	3	4.0	4	2.0	0.730
Q6	42	2	4	4	5.0	5	1.0	<0.001
Q7	42	1	3	4	4.8	5	1.8	=0.001
Q8	42	3	4	5	5.0	5	1.0	<0.001
Q9	42	2	4	4	5.0	5	1.0	<0.001
Q10	42	3	5	5	5.0	5	0.0	<0.001

Table 3: Descriptives of the answers (nonparametric), p-values of the nonparametric Wilcoxon test for one sample for the null hypothesis that the true location of the median is 3 (neutral answer), Q1 = How would you rate the difficulty of acute coagulation management?, Q2 = Acute coagulation management is a complex subject for me., Q3 = I frequently feel time pressure when treating a bleeding patient., Q4 = I regard human factors (such as communication, teamwork, and leadership) essential for successful coagulation management., Q5 = I consider the interpretation of diagnostic coagulation tests (such as ROTEM, lab values) hard to understand., Q6 = I consider acute coagulation management challenging in anticoagulated patients., Q7 = I am under the impression that I lack practice in acute coagulation management., Q8 = I consider precise algorithms and evidence-based guidelines helpful for coagulation management., Q9 = In my daily practice, I usually know the first therapeutic steps in acute bleeding situations and know what to do., Q10 = I consider the availability of resources (e.g., diagnostic test facilities, sufficient staff availability) essential for optimal coagulation management.

3 Differences between the centers

Note that there are many tests in this analysis (10 times Mann-Whitney). For this reason, Bonferroni correction should be applied for the specified p-values.

Variable	Levels	n	Min	q_1	$\widetilde{x}$	q_3	Max	IQR	p-value
Q1	USZ	21	1	2	2	3.0	4	1.0	
	HIBA	21	2	2	3	3.0	4	1.0	
	all	42	1	2	2	3.0	4	1.0	0.08
Q2	USZ	21	2	3	4	4.0	5	1.0	
	HIBA	21	2	3	3	4.0	4	1.0	
	all	42	2	3	4	4.0	5	1.0	0.17
Q3	USZ	21	3	4	4	5.0	5	1.0	
	HIBA	21	1	3	3	4.0	5	1.0	
	all	42	1	3	4	4.8	5	1.8	0.03
Q4	USZ	21	3	4	5	5.0	5	1.0	
	HIBA	21	4	5	5	5.0	5	0.0	
	all	42	3	4	5	5.0	5	1.0	0.15
Q5	USZ	21	2	2	3	4.0	4	2.0	
	HIBA	21	1	2	3	4.0	4	2.0	
	all	42	1	2	3	4.0	4	2.0	0.52
Q6	USZ	21	2	4	4	5.0	5	1.0	
	HIBA	21	3	4	4	4.0	5	0.0	
	all	42	2	4	4	5.0	5	1.0	0.17
Q7	USZ	21	1	3	3	5.0	5	2.0	
	HIBA	21	2	3	4	4.0	5	1.0	
	all	42	1	3	4	4.8	5	1.8	0.87
Q8	USZ	21	4	5	5	5.0	5	0.0	
	HIBA	21	3	4	4	5.0	5	1.0	
	all	42	3	4	5	5.0	5	1.0	0.003
							5	0.0	0.51
Q9	USZ	21	2	4	4	5.0	5	1.0	
	HIBA	21	3	4	4	5.0	5	1.0	
	all	42	2	4	4	5.0	5	1.0	0.64
Q10	USZ	21	3	4	5	5.0	5	1.0	
	HIBA	21	3	5	5	5.0	5	0.0	
	all	42	3	5	5	5.0			

Table 4: Descriptives of the answers by center, p-values for a Mann-Whitney test comparing the answers between USZ and HIBA, USZ = University Hospital Zurich, HIBA = Hospital Italiano de Buenos Aires, Q1 = How would you rate the difficulty of acute coagulation management?, Q2 = Acute coagulation management is a complex subject for me., Q3 = I frequently feel time pressure when treating a bleeding patient., Q4 = I regard human factors (such as communication, teamwork, and leadership) essential for successful coagulation management., Q5 = I consider the interpretation of diagnostic coagulation tests (such as ROTEM, lab values) hard to understand., Q6 = I consider acute coagulation management challenging in anticoagulated patients., Q7 = I am under the impression that I lack practice in acute coagulation management., Q8 = I consider precise algorithms and evidence-based guidelines helpful for coagulation management., Q9 = In my daily practice, I usually know the first therapeutic steps in acute bleeding situations and know what to do., Q10 = I consider the availability of resources (e.g., diagnostic test facilities, sufficient staff availability) essential for optimal coagulation management.

4 Influence of experience

We use median regression to see if the experience of the participants has an influence on their answers. All results were the same: There was no evidence for an influence of the experience. Apart from that, the estimates were close to zero in most cases, so that they would not be clinically relevant.

5 Interrater agreement

Percent agreement between the two raters was 86.15% with a Cohen's Kappa of 0.85, indicating strong agreement between the two raters.

R version and packages used to generate this report

R version: R version 3.6.2 (2019-12-12)

Base packages: stats, graphics, grDevices, utils, datasets, methods, base

Other packages: quantreg, SparseM, irr, lpSolve, dplyr, stringr, reporttools, xtable, ggplot2, knitr

This document was generated on 2020-11-20 at 11:29.
