# Supplementary material for: Physicians’ perceptions regarding acute bleeding management: an international mixed qualitative quantitative study
Source: BMC Anesthesiol. 2021 Feb 10;21:43. doi: 10.1186/s12871-021-01269-x (PMC7874660; doi:10.1186/s12871-021-01269-x)

Step back and reassess – What physicians see as challenges in treating patients with acute bleeding: international, mixed qualitative and quantitative study

Tadzio R. Roche[1], Doreen J. Wetli[1], Julia Braun[2], Ezequiel D. Kataife[3], Federico G. Mileo[3], Donat R. Spahn[1], David W. Tscholl[1,*], Sadiq Said[1]

Additional file 3 – Word cloud, what is easy in coagulation management

Additional file 3. Graphical representation (Wordle.net) of the most common topics in the participants' field notes. The word cloud makes more frequently used words appear larger. This cloud represents the answers to the question: "What do you find easy in coagulation management?".

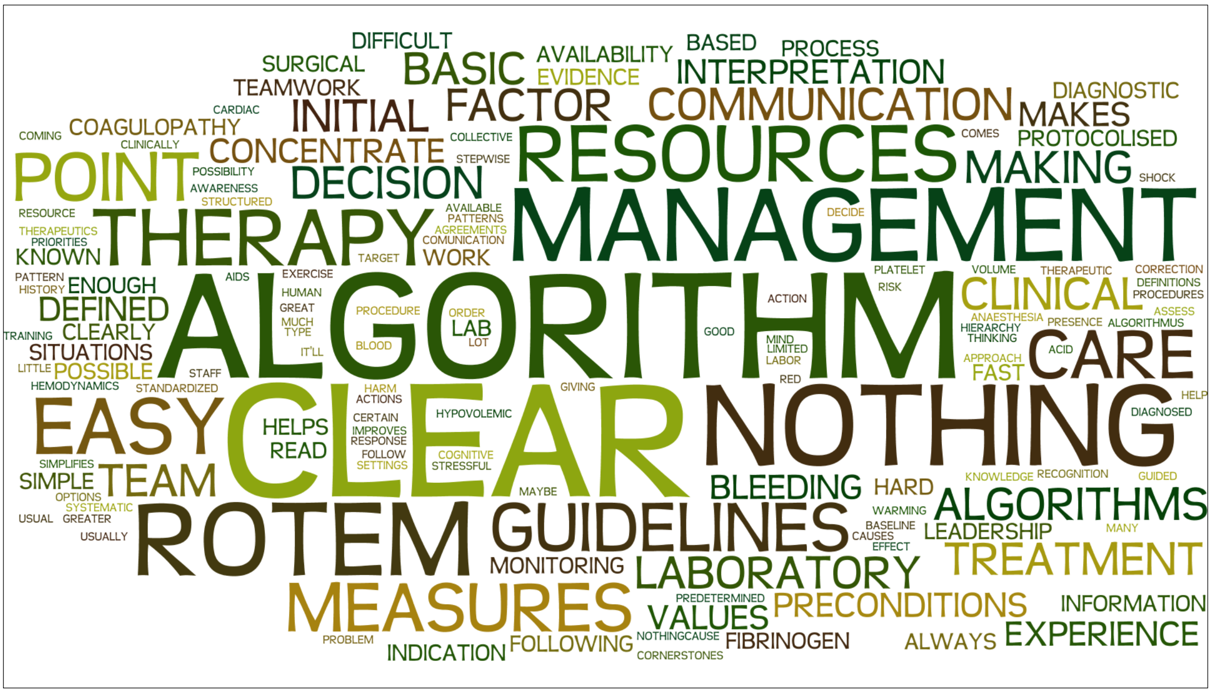

Supplement: Supplementary file 3 — Additional file 3. Graphical representation of the field notes answers to the question: “What do you find easy in coagulation management?”. [file 12871_2021_1269_MOESM3_ESM.pdf]
